# Dosimetric Benefit of Adaptive Magnetic Resonance-Guided Stereotactic Body Radiotherapy of Liver Metastases

**DOI:** 10.3390/cancers14246041

**Published:** 2022-12-08

**Authors:** Fabian Weykamp, Efthimios Katsigiannopulos, Lars Piskorski, Sebastian Regnery, Philipp Hoegen, Jonas Ristau, C. Katharina Renkamp, Jakob Liermann, Tobias Forster, Kristin Lang, Laila König, Carolin Rippke, Carolin Buchele, Jürgen Debus, Sebastian Klüter, Juliane Hörner-Rieber

**Affiliations:** 1 Department of Radiation Oncology, Heidelberg University Hospital, 69120 Heidelberg, Germany; 2 Heidelberg Institute of Radiation Oncology (HIRO), 69120 Heidelberg, Germany; 3 National Center for Tumor Diseases (NCT), 69120 Heidelberg, Germany; 4 Clinical Cooperation Unit Radiation Oncology, German Cancer Research Center (DKFZ), 69120 Heidelberg, Germany; 5 Heidelberg Ion-Beam Therapy Center (HIT), Department of Radiation Oncology, Heidelberg University Hospital, 69120 Heidelberg, Germany; 6 German Cancer Consortium (DKTK), Partner Site Heidelberg, 69120 Heidelberg, Germany

**Keywords:** stereotactic body radiotherapy (SBRT), liver metastases, MR-guided, stereotactic MR-guided adaptive radiotherapy

## Abstract

**Simple Summary:**

Stereotactic body radiotherapy (SBRT) offers a non-invasive treatment approach for patients with inoperable liver metastases. However, conventional cone-beam computed tomography guided radiotherapy only allows for low soft-tissue contrast, which hinders identifying current tumor and surrounding healthy organ positions. The aim of our presented study was to assess dosimetry benefits of stereotactic magnetic resonance (MR)-guided online adaptive radiotherapy (SMART) of liver metastases. Twenty-three patients were treated at the MRIdian Linac. The original irradiation plan was recalculated based on the updated patient anatomy of the day to generate the predicted plan. This predicted plan could then be re-optimized to create an adapted plan. The adapted plan was significantly superior compared to the predicted plan in regard to the tumor treatment dose and the avoidance of high irradiation doses in surrounding healthy organs.

**Abstract:**

(1) Background: To assess dosimetry benefits of stereotactic magnetic resonance (MR)-guided online adaptive radiotherapy (SMART) of liver metastases. (2) Methods: This is a subgroup analysis of an ongoing prospective registry including patients with liver metastases. Patients were treated at the MRIdian Linac between February 2020 and April 2022. The baseline plan was recalculated based on the updated anatomy of the day to generate the predicted plan. This predicted plan could then be re-optimized to create an adapted plan. (3) Results: Twenty-three patients received 30 SMART treatment series of in total 36 liver metastases. Most common primary tumors were colorectal- and pancreatic carcinoma (26.1% respectively). Most frequent fractionation scheme (46.6%) was 50 Gy in five fractions. The adapted plan was significantly superior compared to the predicted plan in regard to planning-target-volume (PTV) coverage, PTV overdosing, and organs-at-risk (OAR) dose constraints violations (91.5 vs. 38.0%, 6 vs. 19% and 0.6 vs. 10.0%; each *p* < 0.001). Plan adaptation significantly increased median BEDD95 by 3.2 Gy (*p* < 0.001). Mean total duration of SMART was 72.4 min. (4) Conclusions: SMART offers individualized ablative irradiation of liver metastases tailored to the daily anatomy with significant superior tumor coverage and improved sparing of OAR.

## 1. Introduction

Stereotactic body radiotherapy (SBRT) offers the selective treatment of liver metastases, which are not amenable to surgery [1,2,3,4]. Applying high tumoricidal irradiation doses whilst simultaneously sparing organs at risk (OAR) requires sophisticated image guidance. However, conventional cone beam CT scans (CBCT) only offer a limited soft tissue contrast [5]. Surrounding abdominal OAR further impede achieving ablative irradiation doses due to high radiosensitivity [6,7,8,9]. Additionally, respiratory motion causes a shift of the liver of up to several centimeters, which in turn can lead to underdosing, if not adequately accounted for [10,11,12,13]. However, dose reduction below 100 Gy BED (alpha/beta = 10 Gy) is associated with inferior local control (LC) [14]. To compensate for respiratory motion, the complete anticipated track of the respective liver lesion during the breathing process (internal target volume) is conventionally irradiated, leading to unnecessary irradiated liver tissue and a potential overlap with critical OAR. On the contrary, surface-guided SBRT operates with intra-fraction monitoring of the body surface. The irradiation can then be applied through gating [15,16,17,18]. However, the lesion itself cannot be directly monitored and deviations of up to 10 mm were described [19]. The Cyberknife system requires implanted fiducials which function as a surrogate structure and are visible on frequent noncoplanar X-ray scans. The irradiation dose is applied through tracking [20]. Again, no direct monitoring of the target lesion is possible, and the placement of fiducials is an invasive procedure. Lately, MR-guided radiotherapy at MR-Linacs has become clinically available. Due to the high soft-tissue contrast, online MR-guided radiotherapy allows for the precise separation of the respective liver lesion and adjacent OAR. Through on-table adaptation the daily anatomy of both the tumor and the OAR can be accounted for [21,22,23]. Moreover, some MR-Linac systems offer gated dose delivery to compensate for respiratory motion and possibly allow to reduce safety margins [24]. Data on this new irradiation device are growing but still scarce. Our first experience with magnetic resonance (MR)-guided SBRT of liver tumors showed promising results in terms of patient reported-outcome. However, online plan adaptation was not implemented at that time [25]. Thus, we sought to evaluate the dosimetry benefit of stereotactic MR-guided online adaptive radiotherapy (SMART) for liver metastases.

## 2. Materials and Methods

We present a subgroup analysis of a prospective observational registry comprising cancer patients with liver metastases. The MR-Linac observational study was approved by the Ethics committee of the University Hospital Heidelberg (S-862/2019). Written informed consent was obtained from all patients, who were included into the study. Patients had refused surgery or the respective liver lesions were deemed inoperable. MR-guided SBRT was carried out at the MRIdian Linac (ViewRay Inc., Mountain View, CA; 0.345 T MRI scanner, 6 MV step-and-shoot intensity modulated radiotherapy) in the Department of Radiation Oncology at Heidelberg University Hospital between February 2020 and April 2022. SBRT was defined as single fraction doses ≥ 4 Gy and with a maximum number of fractions of 12, according to the respective guideline by the working group “Stereotactic Radiotherapy” of the German Society of Radiation Oncology (DEGRO) [26].

### 2.1. Treatment Characteristics

A detailed description of the treatment simulation and planning at our institution was reported earlier [27]. In summary, three-dimensional (3D) simulation MR images, using the TrueFISP sequence (a steady-state coherent MRI sequence) with an acquisition time of 17 s to 25 s, resolutions of either 1.5 × 1.5 mm^2^ or 1.6 × 1.6 mm^2^ and a slice thickness of 1.5–3 mm were obtained in deep inspiration breath-hold (DIBH). Afterwards, a planar cine-MRI in a sagittal plane was carried out to evaluate target motion characteristics [28]. No MRI contrast fluid was used during simulation. However, all patients had an additional diagnostic, contrast-enhanced MRI for treatment planning performed beforehand. Directly after MR simulation, a planning CT scan with and without contrast enhancement was performed in order to gain data on electron density for dose calculation. The gross tumor volume (GTV) comprised the macroscopic tumor volume in the MRI- and CT-images. The GTV was then expanded by 5 mm to obtain the clinical target volume (CTV) respecting surrounding organ borders with a planning target volume (PTV) margin of 3 mm.

Gated dose delivery was performed in DIBH. The respective liver lesion itself was used as the gating structure (region of interest; ROI), if it was visible on the TrueFISP sequence. If not, an anatomical surrogate structure was used as the ROI, which was mainly the adjoining liver surface or a prominent vessel. The ROI was expanded by 3 mm to create the gating boundary. If the ROI left this gating boundary during the gating process (with a tolerance of 3%), the irradiation beam was automatically shut off. This process could be watched by the patients on a monitor.

For daily plan adaptation, the acquired 3D MRI was rigidly co-registered to the original planning MRI with its GTV contours. Afterwards, the OAR contours and the planning CT-imaging were deformably registered to this new MRI. The treating physician then delineated the GTV and checked the OAR contours, which had been adapted by the radiotherapy technologists. Only OAR within the so called PTVexpand were delineated, which was defined as an expansion of the PTV by 1 cm in craniocaudal and 3 cm in circumferential direction [29]. The baseline plan was then recalculated based on the updated anatomy of the day to generate the predicted plan. If dose constraints were violated this predicted plan was re-optimized to create the adapted plan. Afterwards, on-table quality assurance (QA) was performed. All treatment plans were aimed to possess full conformal PTV coverage of at least 95% of the prescribed dose (V100% ≥ 95%) and a determined maximum dose (Dmax) of 125–150%. Fractionation schemes depended on the size of the lesion and its proximity to OAR. If possible, three fractions of 15 Gy were applied (Dmax 150%). Lesions larger than 5 cm were irradiated with eight fractions of 7.5 Gy or five fractions of 10 Gy (each Dmax 125%). Ten fractions of 5 Gy (Dmax 125%) were prescribed if the target lesion was close to abdominal OAR. In one case, homogeneous prescription was used (Dmax 107%). The respective dose constraints for each fractionation scheme are described in the supplements (Supplementary Table S1).

### 2.2. Endpoints, Statistical Methods and Ethics

Dose volume histogram (DVH) parameters as well as target volumes were extracted from the treatment planning system for all fractions of both the predicted and the adapted plans. The biologically effective dose (BED) was calculated assuming an alpha/beta of 10 Gy for liver metastases. Four OAR were chosen for analysis, which were closest to the PTV. The extracted DVH parameters of the predicted as well as the adapted plan of each fraction were compared pairwise. To account for different dose prescriptions, these parameters were normalized. The D95 dose value was normalized to the prescribed dose or 0.95× prescribed dose for homogenous prescriptions. Statistical analysis (IBM SPSS Version 24.0) was performed with the paired Wilcoxon signed-rank test. A significance level of α = 5% was utilized.

## 3. Results

Twenty-three patients received 30 SMART treatment series of in total 36 liver metastases (Table 1).

Most patients suffered from colorectal or pancreatic cancer (respectively 6/26.1%). Median age was 63 years (range 46–89 years) with high performance scores (90%; range 70–100%). Four patients had previous hepatic ablative radiotherapy. Four patients had their SMART divided into two separate treatment series due to a second liver metastasis (Table 2).

One patient had two therapy series two months after the initial treatment series and another therapy series two months thereafter, respectively, due to the development of new liver metastases.

**Figure 1:**
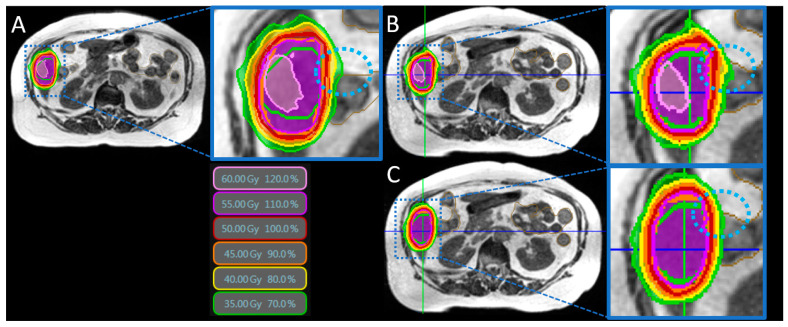
Case study. (**A**) Baseline plan (50 Gy in 10 fractions) with already slightly impaired PTV coverage (91%) due to proximity to the intestines (brown). (**B**) Daily online MRI-guidance revealed that the small bowel had moved slightly forward in ventral direction (dashed light-blue circle) leading to a predicted plan with excessive dose in the small bowel together with impaired PTV coverage (78 %). (**C**) Plan adaption led to the removal of the dose bulge (dashed light-blue circle) and resulted into superior sparing of the small bowel, while simultaneously improving PTV coverage (90%).

Plan adaptation was performed in 200 of the 207 fractions (96.6%) due to OAR violations (case report in Figure 1).

**Figure 2:**
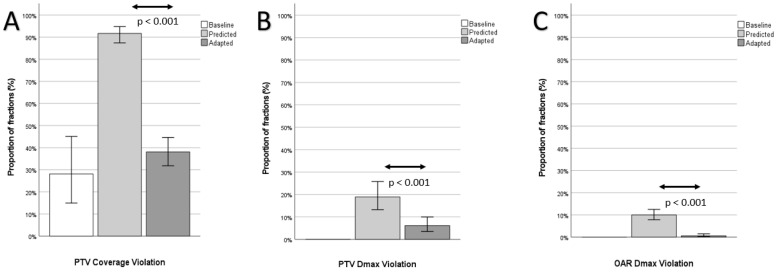
Planning objective violations. Proportion of fractions with one or more violation of the planning target volume (PTV; **A**) coverage, PTV maximum dose (Dmax; **B**) or organ at risk (OAR, **C**) Dmax.

The proportion of fractions with at least one planning objective violation could be significantly decreased with plan adaptation (Figure 2).

**Figure 3:**
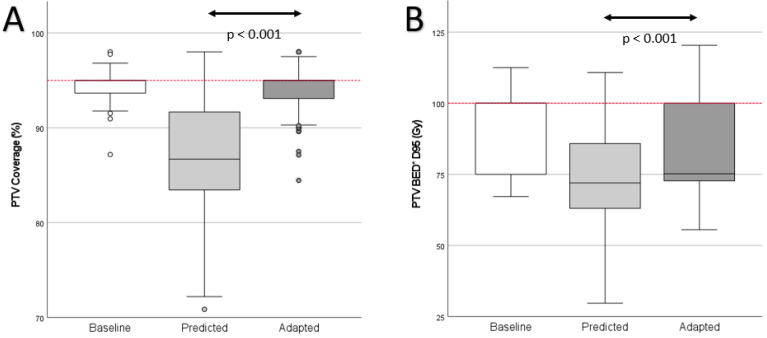
Planning target volume coverage (PTV) and biologically effective dose (BED). (**A**) PTV coverage. The dashed line represents the aimed 95% coverage. (**B**) BED * covering 95% of the PTV (D95). The dashed line represents 100 Gy BED. * α/β = 10 for the liver metastases.

**Figure 4:**
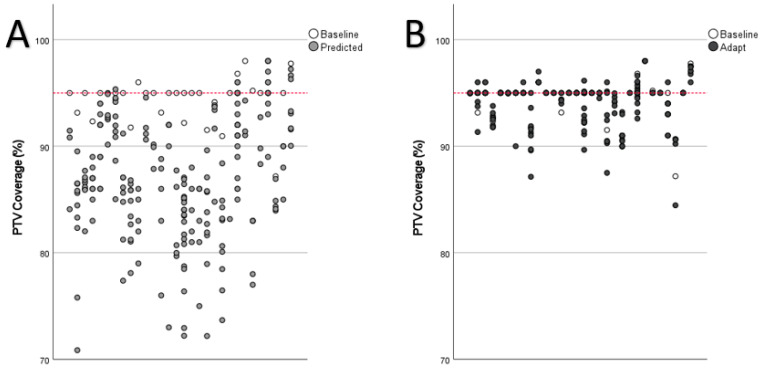
Planning target volume coverage in the predicted plans (**A**) vs. adapted plans (**B**) each in direct comparison with the baseline plan. One verticle column represents one tratment plan. The dashed red line represents the aimed coverage of 95%.

PTV coverage violations were the most common violations and were significantly reduced through plan adaptation (predicted: 183 out of 200, 91.5%, adapted: 76 out of 200, 38.0%, *p* < 0.001; baseline: eight out of 30, 26.6%). Plan adaptation furthermore improved median PTV coverage (Figure 3A and Figure 4; predicted: median 86.7% (range 70.9–98.0%); adapted: 95.0% (range 84.5–98.0%) *p* < 0.001; baseline: 95.0% (range 87.2–98.0%). Plan adaptation significantly increased the median biologically effective dose of the D95 (BED_D95_) by 3.2 Gy (Figure 3B); predicted: 72.0 Gy (range 29.7–110.8 Gy), adapted: 75.2 Gy (range 55.6–120.3 Gy), *p* < 0.001; baseline 100.0 Gy (range 67.2–112.5 Gy).

Overdosing inside the PTV was less frequent in the adapted plans (Figure 2B; predicted: 38 out of 200, 19.0%, adapted: 12 out of 200, 6.0%, *p* < 0.001; baseline: 0/30).

**Figure 5:**
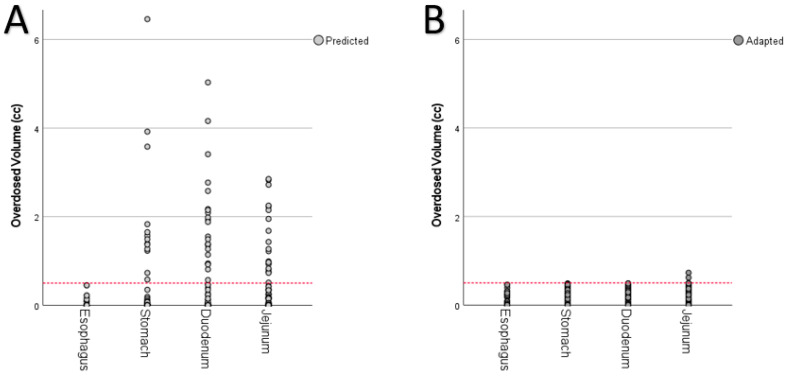
Overdosed volume (cc) of organs at risk for predicted plans (**A**) and adapted plans (**B**). The dashed line represents the aimed threshold of 0.5 cc.

Furthermore, OAR dose constraint violations were significantly reduced through plan adaptation (Figure 2C; predicted: 20 out of 200, 10.0%, adapted: 12 out of 200, 0.6%, *p* < 0.001; baseline: 0 out of 30) as well as the amount of overdosed volume of OAR (Figure 5). In the case of OAR violations, the maximum amount of overdosed volume respectively reached up to 6.5 cc (stomach,), 5.0 cc (duodenum), and 2.9 cc (jejunum) in the predicted plans (Figure 5A) and 0.7 cc (jejunum) in the adapted plans (Figure 5B).

Median PTV volume was 47.4 cc (range 9.7–284.4 cc), with a median PTV change per fraction of 2.5 cc (range 0.1–13.0 cc; Supplementary Figure S1).

## 4. Discussion

In this subgroup analysis of an ongoing prospective registry, we evaluated the dosimetric benefits of SMART in 23 patients with liver metastases. The results fall in line with previously reported dosimetric changes in smaller study populations [30,31,32,33]. PTV coverage was improved through SMART from 87% to 95%. Padgett et al. investigated 10 patients with liver tumors and found 68% of the predicted plans with PTV coverage violations vs. 0% in the adapted plans [30]. Similar to the study presented here, Rogowski et al. evaluated SMART for 11 patients with liver tumors [32]. In the adapted plans, no missing PTV coverage of more than 10% was found. Mayinger et al. investigated 15 patients with liver metastases and found that PTV coverage could be improved by 63% through SMART [31]. High dose SBRT of liver metastases (>100 Gy) is well known to result in better local control than low dose SBRT (≤100 Gy) [34,35]. Hence, a compromised PTV coverage bears the risk of worse oncologic outcome, which can be avoided by SMART.

OAR violations occurred in 6–22% of non-adapted plans and were significantly less frequent in the adapted plans [30,31,32,33]. In one case, the duodenum would have received up to nine times its allowed dose constraint volume [32]. The same accounts for the presented study, where in one case the stomach would have received up to 13 times the allowed dose constraint volume in the non-adapted plan (10 × 5 Gy). This could have led to high grade gastric toxicity. Moreover, in the presented study OAR constraint violations were reduced from 10.0% to 0.6%.

However, one should keep in mind, that plan adaptation is only one component of SMART on the basis of direct MR-imaging of the tumor vicinity during treatment. In a way, MR-guided radiotherapy so far is largely compared with itself, making it more challenging to detect dosimetric benefits. Furthermore, as in the study presented here, patients were included regardless of the tumor localization within the liver. Our currently recruiting MAESTRO study (Magnetic Resonance-guided Adaptive Stereotactic Body Radiotherapy for Hepatic Metastases) investigates the benefit of SMART in liver metastases compared with conventional, CBCT guided SBRT [36]. If high dose SBRT is feasible (>100 Gy BED; alpha/beta = 10 Gy), patients are randomized between SMART and CBCT guided treatment. If not, patients receive upfront SMART in order to evaluate the highest possible dose of SBRT using SMART. The French phase-II RASTAF study (Radiothérapie corporelle stéréotaxique adaptative guidée par IRM des tumeurs du foie) investigates MR-guided dose escalation of liver SBRT: If the lesion is located far from OAR, one additional fraction of 10 Gy will be applied, leading to a total dose of 60 Gy [37].

The MRI can not only be used for image guidance. It is highly sensitive for detecting tissue changes, which directly correlate with the irradiation dose applied to the liver [38,39]. Functional MRI was shown to be predictive for treatment response and therefore offers the potential of future tailored dose prescription through early treatment response assessment during ongoing radiotherapy [40,41]. Furthermore, blood biomarkers, e.g., plasma interleukin-6, were reported to predict local failure after SBRT of liver metastases, paving the way for personalized radiotherapy [42].

## 5. Conclusions

SMART for liver metastases has highly significant dosimetric benefits with increased PTV coverage and reduced maximum doses in OAR. Future studies will evaluate the clinical translation to superior oncologic outcomes as well as the cost effectiveness in order to reveal which patients benefit most from this innovative irradiation technique.

## Figures and Tables

**Table 1:** Patient characteristics (*n* = 23).

median age	63 years	range 46–89 years
median Karnofsky Score	90%	range 70–100%
female/male	11/12	47.8%/52.2%
Primary Tumor		
Colorectal Carcinoma	6	26.1%
Pancreatic Cancer	6	26.1%
Breast Cancer	5	21.7%
Esophageal Cancer	3	13.0%
Renal Cell Carcinoma	1	4.3%
Non Small Cell Lung Cancer	1	4.3%
Parotic Gland Cancer	1	4.3%

**Table 2:** Treatment series characteristics.

Total number of treatment series per patient (*n* = 23 patients)		
*n* = 1	18	78.3%
*n* = 2	4	17.4%
*n* = 4	1	4.3%
Total number of liver metastases per treatment series (*n* = 30 treatment series)		
*n* = 1	24	80.0%
*n* = 2	6	20.0%
Fractionation (*n* = 30 treatment series)		
5 × 10 Gy	14	46.6%
10 × 5 Gy	10	33.3%
3 × 15 Gy	3	10.0%
8 × 7.5 Gy	2	6.7%
12 × 4 Gy	1	3.3%

## Data Availability

The data presented in this study are available on request from the corresponding author.

## Supplementary Materials

The following supporting information can be downloaded at: https://www.mdpi.com/article/10.3390/cancers14246041/s1, Table S1: dose constraints depending on the fractionation; Figure S1: Planning target volume sizes over the treatment courses.
