# Supplementary material for: Dosimetric Benefit of Adaptive Magnetic Resonance-Guided Stereotactic Body Radiotherapy of Liver Metastases
Source: Cancers (Basel). 2022 Dec 8;14(24):6041. doi: 10.3390/cancers14246041 (PMC9775484; doi:10.3390/cancers14246041)
Supplement: Supplementary file 1 [file cancers-14-06041-s001.zip › cancers-2072843-supplementary/Table S1 07122022.pdf]

Table S1. Dose constraints depending on the fractionation.

	**3 fx**	**5 fx**	**8 fx**	**10 fx**
esophagus 0.5cc	<25.2 Gy	<34 Gy	<40 Gy	<43.5 Gy
stomach/intestine 0.5cc	<22.2 Gy	<35 Gy	<40 Gy	<43.5 Gy
liver minus CTV ≥700cc	<19.2 Gy	<24 Gy	<29 Gy	<32 Gy
kidney mean dose	<8.5 Gy	<10 Gy	<11.5 Gy	<12 Gy
spinal cord 0.1cc	<21.6 Gy	<27 Gy	<32 Gy	<35 Gy
heart 0.5cc	<26 Gy	<29 Gy	<60 Gy	<66 Gy

All dose constraints were (strict) minimum requirements. Whenever possible, lower thresholds were aimed for. This allowed for mild constraint violations in rare cases, where otherwise a sufficient target dose would not have been possible.
