# Supplementary figures and images for: Dosimetric Benefit of Adaptive Magnetic Resonance-Guided Stereotactic Body Radiotherapy of Liver Metastases
Source: Cancers (Basel). 2022 Dec 8;14(24):6041. doi: 10.3390/cancers14246041 (PMC9775484; doi:10.3390/cancers14246041)

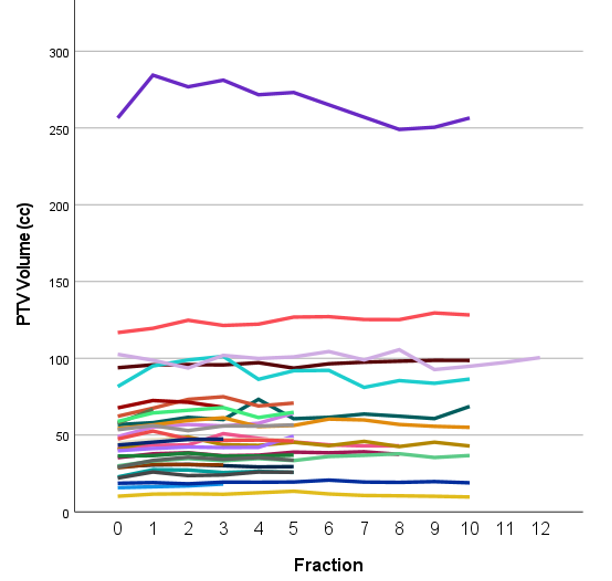

Supplement: Supplementary file 1 [file cancers-14-06041-s001.zip › cancers-2072843-supplementary/Figure S1 - 07122022.PNG]
